# Factors Associated with Uptake of Intermittent Preventive Treatment for Malaria During Pregnancy. Analysis of Data from the Tanzania 2015-2016 Demographic Health Survey and Malaria Indicator Survey

**DOI:** 10.24248/eahrj.v6i2.692

**Published:** 2022-11-30

**Authors:** Theresia J. Masoi, Fabiola V. Moshi, Maximilian B. Tungaraza

**Affiliations:** a Department of Clinical Nursing, Dodoma, Tanzania; b Department of Nursing education and Management, Dodoma, Tanzania

## Abstract

**Background:**

Malaria is a life-threatening disease caused by parasites that are transmitted to people through bites of infected female Anopheles mosquitoes. Africa is the home to over 90% of malaria burden when compared to other regions of the world. The region is estimated to have a dominance of 94% of maternal deaths occurring in the world. The purpose of this study was to identify factors associated with the uptake of IPTp-SP among pregnant women in Tanzania. Method: The study used data from the 2015-16 Tanzania Demographic and Health Survey and Malaria Indicators Survey (2015-16 TDHS-MIS). A total of 6,885 women of active reproductive age from 15 to 49 were included in the analysis. Both univariate and multiple regression analyses were performed to determine factors associated with uptake of IPTp-SP during pregnancy in Tanzania.

**Results:**

A total of 4764(68.6%) of pregnant women took at least one dose of IPTp-SP during Antenatal Care (ANC) visits. After adjusting for confounders, factors which were associated with uptake of IPTp-SP were; early antenatal booking, (AOR=1.495 *p<.001*); age group of pregnant woman [20 to 34 years (AOR=1.446, *p=.001*), more than 34 years (AOR=1.648, p<.001)]; wealth index [middle (AOR=1.418, p<.001), rich (AOR=1.589, *p<.001*)], education level [primary education (AOR=1.457, p<.001), secondary education AOR=1.653, p<.001]; parity [para 2 to 4 (AOR=1.213, *p=.014*), para 5 and above (AOR=1.226, *p=.043*)] and zone [Mainland rural (AOR=0.647, *p=.019*), Unguja (AOR=0.172, *p<.001*) and Pemba (AOR=0.310, *p<0.001*)].

**Conclusion:**

Factors associated with uptake of IPTp-SP during pregnancy were; timing for ANC booking, age of pregnant woman, parity, level of education, and place of residence.

## BACKGROUND

Malaria is a life-threatening disease caused by parasites that are transmitted to people through bites of infected female Anopheles mosquitoes.^1^ Malaria is a preventable and curable disease.^2^ Africa is the home to over 90% of malaria burden when compared to other regions of the world.^2^ The region is estimated to have a dominance of 94% of maternal deaths occurring in the World.^4^

Malaria is one of the indirect causes of maternal deaths, accounting for about 15% of all reported maternal deaths in the world. In 2015, malaria was considered the third cause of deaths among women of child bearing age in the world. About 24 million pregnant women in the sub-Saharan Africa are affected with malaria every year. In endemic areas, events of maternal deaths due to malaria accounts for about 25%.^6^ A study that used District Health Information System (DHIS) data of 2014 and 2017 in Tanzania, reported malaria prevalence of 8.1% and 6.7% respectively, indicating an absolute difference of 1.4.%^7^

Primigravidae, adolescents, and pregnant women coinfected with Human Immunodeficiency Virus (HIV)/Acquired Immunodeficiency Syndrome (AIDS) are usually accompanied with adverse complication.^6^ Commonly reported compactions of malaria in pregnancy include; placental malaria, maternal anaemia, abortions, premature labour, intrauterine growth retardation and low birth weight.^8^ Asymptomatic malaria is also common among primigravidae especially among those from malaria-endemic areas.^9^ Maternal decline of immunity pronounced during the first and second pregnancies predispose them to high vulnerability to malaria^10^.

The World Health Organization (WHO) and the Global Malaria Community targets a malaria free world by 2030.^2^ Nurses should routinely administer Intermittent Preventive Treatment of Malaria in Pregnancy Sulfadoxine-Pyrimethamine (IPTp-SP) to all pregnant women attending antenatal care services as one of the strategies to preventing malaria in pregnancy. Administration of IPTp-SP is based on the assumption that every pregnant woman living in malaria-endemic areas has malaria parasites in her blood or placenta, regardless of whether she is symptomatic or asymptomatic.^10^ The appropriate time to administer the first IPTp-SP dose is during the early second trimester. Subsequent doses are scheduled at 4 weeks apart while the last dose can be administered during late pregnancy, i.e. after 36 weeks of gestation, up to the time of delivery. Frontline antenatal care providers should ensure that pregnant women receive not less than 3 doses of IPTp-SP before delivery.^11^

According to Tanzania Demographic and Health Survey and Malaria Indicator Survey (TDHS-MIS) (2015/2016), 71% of pregnant women who attended antenatal care services in Tanzania received IPTp-SP.^12^ The *2019 World Malaria Report* indicated that Tanzania and Burkina Faso had over two-third of their pregnant women receive 3 doses of Intermittent Preventive Treatment of Malaria in Pregnancy (IPTp3) in 2018. The report also showed low coverage (31%) of IPTp-SP across sub-Saharan Africa.^13^

Several factors have been raised as enablers and/or barriers to IPTp-SP uptake. A study conducted in Korogwe District, Tanzania showed that nurses do not provide IPTp-SP as Direct Observed Therapy (DOT) due to reasons such as shortage of clean drinking water; therefore, it is not clear whether the provided anti-malaria drugs are taken as intended^14^. Having knowledge on when to start and stop IPTp-SP can also be a challenge to IPTp-SP uptake.^15,16^ Studies have also revealed that factors such as education level of up to college level, early booking and having atleast 4 ANC visits contribute to adequate uptake.^16,17^ Moreover, having parity of at least 4, being single and self-employment have been reported to impede the uptake.^18^ The nurse's limited knowledge on IPTp-SP protocol was a barrier to adequate uptake of the service as it was reported in Ghana.^19^ Similarly, IPTp-SP uptake can be determined by the age of the woman. Several studies from elsewhere showed that as the woman's age increases, the likelihood to IPTp-SP uptake also increases.^17,20,21^.

The Tanzanian government has invested in ensuring that all pregnant women access IPTp-SP services during their visit for Antenatal Care Services. The government not only expanded health facilities to each village but also deployed adequate number of nurse/midwives to ensure pregnant women receive skilled services. However, according to TDHS-MIS (2015/2016), 24% and 49% of pregnant women book ANC services late and had inadequate ANC visits respectively.^12^ This can impede the pregnant women to receive IPTp-SP timely. Therefore, this study aimed at analysing factors associated with uptake of intermittent preventive treatment for malaria dose at least once during pregnancy using data from TDHS-MIS.

## METHOD

### Study Area and Period

The study was conducted in the United Republic of Tanzania from August 22, 2015, through February 14, 2016. Tanzania is among the countries found in East Africa. It is the largest country within East Africa and it covers 940,000 square kilometres with 60,000 square kilometres of inland water. The country lies south of the equator and shares borders with 8 countries: Kenya and Uganda to the North; Rwanda, Burundi, the Democratic Republic of Congo, and Zambia to the West; and Malawi and Mozambique to the South.

### Study Design

This was a national-based cross-sectional study utilising the 2015/2016 Tanzania Demographic and Health Survey and Malaria Indicator Survey (TDHS-MIS) dataset.

### Study Population

All women of reproductive age (15 to 49 years) were eligible to participate in the study. The study used Individual file recode (TZIR7BFL) with a total of 13,266 women who responded to the survey (97% response rate). The study included only women who responded to the question on if they have ever taken anti-malaria medication during pregnancy. Those who were not able to recall the timing and those who did not respond to the question were excluded from the analysis. A total of 6,885 women who had given birth within 5 years preceding the survey were included in the study.

Two stages of sampling were used to obtain sample data for urban and rural areas in Tanzania Mainland and Zanzibar. In the first stage, a total of 608 clusters were selected and in the second stage, a systematic selection of households was adopted. A total of 22 households were systematically selected from each cluster, yielding a representative probability sample of 13,376 households for the 2015-16 TDHS-MIS. To enhance representativeness, Tanzania was divided into 9 geographic zones. Regions were grouped into zones so as to reduce sampling error by way of increasing the number of people in the denominator. The zones were; western zone (Tabora and Kigoma regions), Northern zone (Kilimanjaro, Tanga, and Arusha), Central zone (Dodoma, Singida and Manyara), Southern Highland zone (Iringa, Njombe, and Iringa), Southern zone (Lindi and Mtwara), South West Highland zone (Mbeya Rukwa and Katavi), Lake zone (Kagera, Mwanza, Geita, Mara, Simiyu, and Shinyanga), Eastern zone (Dar es Salaam, Pwani, and Morogoro) and Zanzibar zone (Kaskazini Unguja, Kusini Unguja, Mjini Magharibi, Kaskazini Pemba and Kusini Pemba).

### Study Variables

Literature review was done and a conceptual framework was developed to guide the data review process. The conceptual framework defined the independent variables (socio-demographic and obstetric characteristics of a woman) and the dependent variables (having ever taken anti-malaria drugs during pregnancy), coded 1 for those who have ever taken anti-malaria drugs during pregnancy and 0 for those who had never.

### Data Collection Tools

The 2015-16 TDHS-MIS used household and individual questionnaires. These questionnaires were based on the Demographic and Health Surveys (DHS) standard AIDS Indicator Survey and Malaria Indicator Survey questionnaires standards. They were adapted and modified to reflect the Tanzanian population. They were translated into Kiswahili, Tanzania's National language.

The data presented in this study is from the individual questionnaires.

### Data Analysis

Data was analysed using IBM SPSS version 20. Women who took anti malaria drugs at least once were coded as 1 and those who never took anti malaria drugs during pregnancy were coded 0. Data analysis started by describing all study variables using frequencies and percentages. The association between dependent and independent variables was assessed using the chi-squared test. Univariate, binary and multivariable analysis was performed for descriptive and significant predictors of uptake of IPTp-SP. All analyses were based at a 5% level of significance.

### Ethics Approval and Consent to Participate

Data collection and the survey content and protocol were approved by Tanzania's National Institute for Medical Research (NIMR), the Zanzibar Medical Ethics and Research Committee (ZAMREC), the Institutional Review Board of ICF International, and the Centres for Disease Control and Prevention in Atlanta, USA. Participants provided verbal consents and the household interviews took place privately. For participants under the age of 18, written consent was requested from their parent or guardian.

## RESULTS

### Socio-demographic Characteristics

The study included 6,885 women of reproductive age who had given birth within 5 years preceding the survey. Majority of study respondents 5,113(73.8%) resided in the rural setting of Tanzania, 4,557(65.8%) were aged 20 to 34 years. 4,209(60.8%) had primary education, and 5,650(86.1%) were married (Table 1).

**TABLE 1:** Socio-Demographic Characteristics of Respondents

Variables	Frequency	Percent (%)
Place of residence		
Urban	1811	26.2
Rural	5113	73.8
Age group		
Less than 20 years	541	7.8
20 to 34 years	4557	65.8
More than 34 years	1826	26.4
Educational level		
No education	1329	19.2
Primary education	4209	60.8
Secondary	1326	19.2
Higher	60	0.9
Parity		
Para one	1595	23
Para 2-4	3154	45.6
Para 5+	2175	31.4
Wealth index		
Poor	2734	39.5
Middle	1363	19.7
Rich	2827	40.8
Marital Status		
Never in union	441	6.4
Married	5650	86.1
Widow	119	1.7
Separated	714	10.3
Respondent currently working		
Not working	1498	21.6
Working	5426	78.4
Mainland/Zanzibar		
Mainland urban	1618	23.4
Mainland rural	4357	62.9
Unguja (Zanzibar Island)	594	8.6
Pemba (Pemba Island)	355	5.1

**FIGURE 1:**
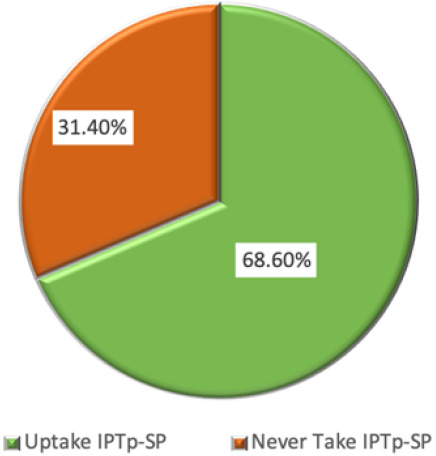
Uptake of SP/Fansidar Atleast Once During Pregnancy

Uptake of anti-malaria at list once during pregnancy A total of 4,725(68.6%) took anti malaria prophylaxis at least once during their pregnancy while a total of 2,160 (31.4%) never took (Figure 1).

### The Relationship between Women's Characteristics and Uptake of Malaria Drugs for Intermittent Treatment of Malaria during Pregnancy

Variables which showed significant relationship were; place of residence (X^2^=70.107 and *p<.001*), age group (X^2^=33.932 and *p<.001*), education level (X^2^=70.342 and p<.001), parity (X^2^=8.395 and *p=.015*), timing for ANC visits (X^2^=66.16 and *p<.001*), Number of ANC visits (X^2^=78.962 and *p<.001*), wealth index (X^2^=70.107 and *p<.001*) and zones (X^2^=330.254 and *p<.001*) (Table 2).

**TABLE 2:** The Relationship between Women's Characteristics and uptake of Malaria drugs for Intermittent Treatment of Malaria during Pregnancy

Variable	Uptake of anti-malaria		X2	p-value
	Never n(%)	at least once n(%)		
Age categories of respondents			33.932	<0.001
15years-24years	778 (35.9)	1389 (64.1)		
25years-29years	476 (29.6)	1133 (70.4)		
30years-34years	354 (27.3)	945 (72.7)		
35years-49years	552 (30.5)	1258 (69.5)		
Type of place of residence			128.4	<0.001
Urban	373 (20.7)	1427 (79.3)		
Rural	1787 (35.1)	3298 (64.9)		
Wealth Index			70.107	<0.001
Poor	1007 (37)	1716 (63)		
Middle	405 (29.8)	952 (70.2)		
Rich	748 (26.7)	2057 (73.3)		
Marital Status			6.586	0.086
Never in union	118 (26.9)	321 (73.1)		
Living with partner	1799 (32)	3824 (68)		
Widowed	37 (31.1)	82 (68.9)		
Divorced	206 (29.3)	498 (70.7)		
ANC Visits			78.962	<0.001
Adequate	916 (26.4)	2549 (73.6)		
Inadequate	1244 (36.4)	2176 (63.6)		
Parity of the respondent			8.395	0.015
Para one	524 (33)	1064 (67)		
2 to 4	929 (29.6)	2209 (70.4)		
Para 5+	707 (32.7)	1452 (67.3)		
Timing for ANC Booking			66.16	<0.001
Late booking	1796 (33.9)	3509 (66.1)		
Early booking	364 (23)	1216 (77)		
Highest educational level			70.342	<0.001
No education	537 (40.7)	783 (59.3)		
Primary	1195 (28.5)	2998 (71.5)		
Secondary	413 (31.5)	899 (68.5)		
Higher	15 (25)	45 (75)		
Mainland/Zanzibar			330.254	<0.001
Mainland urban	279 (17.3)	1333 (82.7)		
Mainland rural	1404 (32.3)	2937 (67.7)		
Unguja (Zanzibar Island)	318 (55)	260 (45)		
Pemba (Pemba Island)	159 (44.9)	195 (55.1)		

### Factors Associated with Uptake of Malaria Drugs for Intermittent Treatment of Malaria during Pregnancy

After adjusted for confounders, factors which were booking, (AOR=1.495 at 95% CI=1.306-1.712, *p<.001*); age group of pregnant woman [20 - 34 years (AOR=1.446 at 95% CI= 1.1690-1.787, *p=.001*), above 34 years (AOR=1.648 at 95% CI=1.270-2.137, *p<.001*)] less than 20 years was a reference population; wealth index [middle (AOR=1.418 at 95% CI=1.226-1.641, *p<.001*), rich (AOR=1.589 at 95% CI=1.352-1.866, *p<.001*)] poor was a reference population, education level [primary education (AOR=1.457 at 95% CI=1.271-1.670, *p<.001*), secondary education AOR=1.653 at 95% CI=1.354-1.866, *p<.001*], no formal education was a reference population; parity [para 2 to 4 (AOR=1.213 at 95% CI=1.04-1.414, *p=.014*), para 5 and above (AOR=1.226 at 95% CI=1.006-1.493, *p=0.043*)], para one was a reference population and zone [Mainland rural (AOR=0.647 at 95% CI=0.45-0.93, *p=0.019*), Unguja (AOR=0.172 at 95% CI=0.123-0.241, *p<.001*) and Pemba (AOR=0.310 at 95% CI=0.211-0.456, *p<.001*)] (Table 3).

**TABLE 3:** Factors Associated with Uptake of Anti-Malarial Dose atleast Once During Pregnancy

Variable	OR	95%CI		p-value	AOR	95%CI		p-value
		Lower	Upper			Lower	Upper	
ANC Booking								
Late booking	1				1			
Early booking	1.702	1.495	1.939	<0.001	1.495	1.306	1.712	<0.001
Age groups								
Less than 20 years	1				1			
20 to 34 years	1.62	1.35	1.944	<0.001	1.446	1.169	1.787	<0.001
More than 34 years	1.631	1.338	1.988	<0.001	1.648	1.27	2.137	<0.001
Place of residence								
Urban	1				1			
Rural	0.483	0.425	0.548	<0.001	0.956	0.688	1.328	0.788
Wealth index								
Poor	1				1			
Middle	1.379	1.199	1.586	<0.001	1.418	1.226	1.641	<0.001
Rich	1.621	1.446	1.817	<0.001	1.589	1.352	1.866	<0.001
Educational level								
No education	1				1			
Primary education	1.71	1.504	1.944	<0.001	1.457	1.271	1.67	<0.001
Secondary	1.499	1.278	1.758	<0.001	1.653	1.354	2.018	<0.001
Higher	2.034	1.122	3.686	0.019	1.456	0.758	2.796	0.260
Parity								
Para one	1				1			
Para 2-4	1.172	1.029	1.334	0.016	1.213	1.04	1.414	0.014
Para 5+	1.016	0.885	1.166	0.822	1.226	1.006	1.493	0.043
Mainland/Zanzibar								
Mainland urban	1				1			
Mainland rural	0.438	0.38	0.506	<0.001	0.647	0.45	0.93	0.019
Unguja (Zanzibar Island)	0.181	0.147	0.222	<0.001	0.172	0.123	0.241	<0.001
Pemba (Pemba Island)	0.257	0.201	0.328	<0.001	0.31	0.211	0.456	<0.001

## DISCUSSION

A national-based cross-sectional study utilising the 2015-16 TDHS-MIS dataset was carried out among women of reproductive age (aged 15 to 49 years) to investigate factors associated with uptake of IPTp-SP in Tanzania. The outcome of the survey revealed that early ANC booking increased the odds of IPTp-SP uptake to more than one-fold compared to reference category. This finding mirrors the findings of a study conducted in Ghana, which also revealed early ANC booking being among the factor which influenced IPTp-SP uptake.^19^ Significance similarity was also reported in a study conducted in Arusha, Tanzania which revealed that early booking increased the odds of IPTp-SP uptake to almost two-folds.^16^ Early booking provides the pregnant women with ample chance to have adequate ANC visits and hence increasing the chance of IPTp-SP uptake. The research evidence support the premise that increased ANC visits increased the probability to IPTp-SP uptake.^17–20^

This study revealed that maternal age had a positive influence to IPTp-SP uptake. The uptake of IPTp-SP increases with increasing maternal age. This results echoed the DHS of 2013 which indicated that women with greater than or equal to 30 years had increased odds to IPTp-SP uptake.^21^ Contrary to this, a demographic health survey (UDHS-2015/2016) conducted in Uganda indicated that maternal age did not contribute to IPTp-SP uptake among pregnant women.^17^ Similarly, the results of a systematic and meta-analysis study conducted in sub-Saharan Africa demonstrated insignificant association between maternal age and IPTp-SP uptake.^20^ Differing socio-demographic characteristics of the respondents could be the reason for the demonstrated conflicting results.

The analysis also reported wealth index of the respondents to be a significant determinant of IPTp-SP uptake. The likelihood for IPTp-SP use increased based on the woman's wealth index. Middle class and richer women were more than one-fold likely to have adequate IPTp-SP associated with uptake of IPTp-SP were; early ANC uptake compared to the poor group. This observation is in line with what was revealed from a data analysis study conducted in Nigeria.^20^ Being wealthy implies that the pregnant woman has the ability to cater for costs relating to access and utilisation of ANC services, and hence, promoting IPTp-SP uptake. There is a positive relationship between increasing wealth quintile and receiving ANC services which increases the chance of pregnant women to received IPTp-SP services.

The uptake of IPTp-SP was also found to increase with increasing education level of the woman. Pregnant women with primary education were one-fold versus those with secondary education or above whose odds were almost two-folds likely to have adequate IPTp-SP uptake compared to the reference category. This observation is supported by several other studies conducted in sub-Saharan Africa.^17,20^ Educated women tend to have greater awareness and knowledge of the existence of IPTp-SP services and the benefits of using such services.^22^

This study showed that multi-parity and grand-multi-parity increased the likelihood of uptake of IPTp-SP compared to primiparity. However, this observation is contradicted by a cross-sectional study conducted in Ghana at Winneba, Trauma and Orthopedic Hospital, which revealed that women with 4 and above parities had the least likelihood to use IPTp-SP during pregnancy.^18^ The difference in the findings can be attributed to the different sample sizes used, 6,885 and 391. A similar study conducted in Ethiopia reported the significant association maternal parity and health services utilisation including IPTp-SP uptake.^23^

It was further revealed that being a resident of Mainland rural, Unguja and Pemba was negatively associated with IPTp-SP uptake compared to urban Mainland residency. The observed results could be due to socio-cultural differences especially with reference to residents of Unguja and Pemba Islands. Research evidence have linked rural residency with illiteracy, inadequate access to health services, inaccessible to health information updates, poverty etc., and all these attribute to inadequate IPTp-SP uptake.^24,25^

## CONCLUSION

Although IPTp-SP is a recommended routine measure against malaria infection during pregnancy, a considerable percentage of pregnant women do not receive the service. Based on this study, women who are most likely not to receive IPTp-SP are; those who book late for their ANC, below 20 years of age, with no formal education, low parity and reside in Mainland rural, Island Unguja and Pemba. Innovative strategies are recommended to address the challenge of low uptake of IPTp-SP during pregnancy.
